# Isoliquiritigenin, a potent human monoamine oxidase inhibitor, modulates dopamine D_1_, D_3_, and vasopressin V_1A_ receptors

**DOI:** 10.1038/s41598-021-02843-6

**Published:** 2021-12-07

**Authors:** Ritu Prajapati, Su Hui Seong, Se Eun Park, Pradeep Paudel, Hyun Ah Jung, Jae Sue Choi

**Affiliations:** 1 Department of Food and Life Science, Pukyong National University, Busan, 48513 Republic of Korea; 2 Division of Natural Products Research, Honam National Institute of Biological Resource, Mokpo, 58762 Republic of Korea; 3 Department of Biomedical Science, Asan Medical Institute of Convergence Science and Technology, Seoul, 05505 Republic of Korea; 4 National Center for Natural Products Research, Research Institute of Pharmaceutical Sciences, The University of Mississippi, Oxford, MS 38677 USA; 5 Department of Food Science and Human Nutrition, Jeonbuk National University, Jeonju, 54896 Republic of Korea

**Keywords:** Biotechnology, Computational biology and bioinformatics, Drug discovery, Plant sciences

## Abstract

Isoliquiritigenin (= 4,2′,4′-Trihydroxychalcone) (ILG) is a major constituent of the Glycyrrhizae Rhizoma that has significant neuroprotective functions. In the present study, we re-examined the potential of ILG to inhibit human monoamine oxidase (hMAO) in vitro and established its mechanism of inhibition through a kinetics study and molecular docking examination. ILG showed competitive inhibition of hMAO-A and mixed inhibition of hMAO-B with IC_50_ values of 0.68 and 0.33 µM, respectively, which varied slightly from the reported IC_50_ values. Since ILG has been reported to reduce dopaminergic neurodegeneration and psychostimulant-induced toxicity (both of which are related to dopamine and vasopressin receptors), we investigated the binding affinity and modulatory functions of ILG on dopamine and vasopressin receptors. ILG was explored as an antagonist of the D_1_ receptor and an agonist of the D_3_ and V_1A_ receptors with good potency. An in silico docking investigation revealed that ILG can interact with active site residues at target receptors with low binding energies. These activities of ILG on hMAO and brain receptors suggest the potential role of the compound to ameliorate dopaminergic deficits, depression, anxiety, and associated symptoms in Parkinson’s disease and other neuronal disorders.

## Introduction

Neurodegenerative diseases (NDDs) include a heterogeneous set of disorders characterized by progressive and irreversible damage to the neurons of the central nervous system (CNS) that can lead to functional and mental impairments^1^. Alzheimer’s disease (AD) and Parkinson’s disease (PD) are the common neurodegenerative disorders; each has a prevalence of approximately 1% of the elderly population in their late sixties and even more in the latter decades of life^2^. PD is a nervous system disorder characterized by progressive degeneration of nigrostriatal dopaminergic neurons in the midbrain, resulting in depletion of striatal dopamine (DA). Motor symptoms (such as tremor at rest, rigidity, bradykinesia, and postural instability) are the hallmarks of PD and often are accompanied by non-motor features, like cognitive dysfunction, depression, anxiety, and other socio-behavioral anomalies. Multiple mechanisms that provoke neurodegeneration have been proposed, such as complex molecular changes involving DA and non-dopamine neurons (such as cholinergic and gamma-aminobutyric acid-B [GABA]ergic neurons) and non-neuronal cells (such as microglia and astrocytes), perturbed cell functions like mitochondrial dysfunction resulting from excessive reactive oxygen species (ROS) or either underexpression or mutation of protective genes like peroxisome proliferator-activated receptor-gamma activator-1α (PGC-1α) and parkin-associated proteins (PARK6, PARK7, and PARK8). In addition, α-synuclein aggregation is a pathological marker of PD. Overall, the pathogenesis of PD represents a complex multifactorial process involving neurodegeneration and progression of the disease^3,4^.

For multifactorial diseases like PD that have intertwined pathophysiological routes, the “one gene, one target, one drug” paradigm might be inadequate to achieve the required therapeutic effect. Therefore, multi-target treatment strategies like combination therapy (also known as cocktail-drug therapy) that combines two or more drugs with an independent mechanism of action to obtain either an added or a synergistic effect are common. For instance, levodopa (l-Dopa) often is combined with a DA receptor agonist, a monoamine oxidase-B (MAO-B) inhibitor, or a catechol-*O*-methyltransferase inhibitor to allay motor symptoms in advanced PD. Such combination nevertheless holds drawbacks, such as polypharmacology-related adverse drug reactions due to varying pharmacokinetic and pharmacodynamics profiles of each drug and medication non-adherence and noncompliance. Thus, another multi-target strategy in which a single chemical entity can influence more than one target has piqued the interest of medicinal chemists. This approach can avoid adverse effects associated with combination drugs and attain a more predictable pharmacokinetic profile of a single drug compared to the multiple drugs administered in combination therapy^3,5^.

**Figure 1:**
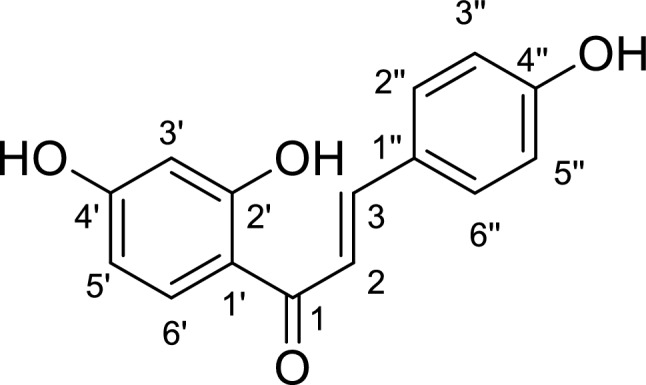
The chemical structure of isoliquiritigenin*.*

Licorice is an ancient medicinal herb constituting three main species: *Glycyrrhiza glabra* L., *Glycyrrhiza uralensis* Fish. ex DC., and *Glycyrrhiza inflata* Batalin. Licorice (Glycyrrhizae Rhizoma) possess multiple pharmacological activities (such as antioxidant, anti-inflammatory, anti-diabetic, anti-cancer, and memory-enhancing effects) owing to the constitution of a variety of bioactive constituents including chalcones (isoliquiritin, isoliquiritin apioside, licuraside, isoliquiritigenin, and licochalcone A), isoflavonoids (licoricidin and glabridin), flavanones (liquiritin, liquiritin apioside, and the estrogenic liquiritigenin), the prenyl flavanoid glycycoumarin, the triterpene glycyrrhetinic acid, and the saponin glycyrrhizin^6,7^. Of these, isoliquiritigenin (ILG) (= 4,2′,4′-trihydroxychalcone) represents one of the most pharmacologically important components of the *Glycyrrhiza* root and its chemical structure is presented in Fig. 1. It has exhibited significant antiproliferative activity on different cancer cells, along with anti-inflammatory, hepatoprotective, cardioprotective, antiangiogenic, antimicrobial, immunoregulatory, neuroprotective, and diabetic complication-preventing effects^8–10^. Recently, multiple studies have explored the promising effects of ILG as a neuroprotective and neurorescueing compound through inhibition of intracellular ROS generation; antioxidative action; attenuation of synaptic dysfunction, neuronal damage, and neuroinflammation, confirming its usefulness in neurodegenerative diseases^11–14^.

Monoamine oxidases (MAOs) are the principal flavoproteins responsible for the catalytic breakdown of monoamine neurotransmitters, and two isoforms of MAO (MAO-A and MAO-B) are present in human tissues. Serotonin and norepinephrine are selectively metabolized by MAO-A, whereas phenylethylamine and benzylamine are preferentially degraded by MAO-B. DA, tyramine, and tryptamine are deaminated by both forms of MAOs^15^. Thus, the inhibitors of MAO can be used as both prophylactic and therapeutic agents in neurodegenerative disorders, including AD, PD, anxiety, depression, and schizophrenia, where a loss of monoamines is observed^16–19^. Earlier studies have shown that ILG can inhibit MAOs, however, its inhibitory potential varies according to animal models. ILG displayed a weak inhibition of MAO from bovine serum (50% inhibition of MAO activity at concentrations > 200 µM)^20^. Whereas, ILG isolated from *Sinofranchetia chinensis* demonstrated significant inhibition of rat brain MAO-A and MAO-B (rMAO-A/B), with IC_50_ values of 13.9 and 47.2 µM, respectively^21^. ILG inhibited human MAO-A (hMAO-A) and human MAO-B (hMAO-B), with IC_50_ values 41.5 and 21.8 µM, respectively^22^.

A study by Zhuo et al*.* on the hMAO inhibitory activities of ILG derivatives found that ILG is selective toward hMAO-B, in contrast to higher selectivity toward rMAO-A. The synthesized compound **C8** showed higher potency and selectivity for hMAO-B than ILG; the enzymatic activity of **C8** against hMAO-B was about 16 times greater than that of ILG. However, molecular docking showed greater proximity of ILG toward the catalytic site active residues of hMAO-B than of **C8**^22–24^. To examine the incongruent results between the enzymatic activity and the in silico docking study and to investigate the reproducibility of the inhibitory action of ILG on recombinant hMAOs, we re-evaluated the functions of ILG on hMAO-A and hMAO-B using a homogenous luminescent assay, enzyme kinetics, and molecular simulation studies. The present work includes a detailed study of inhibition kinetics and an in silico docking study focusing on the ILG-hMAO (A/B) complex, which has not been reported.

ILG was found to suppress cocaine-induced DA release in the rat brain by modulating the gamma-aminobutyric acid-B (GABA_B_) receptor^25^ and also, inhibit N-methyl-d-aspartate receptor-induced Ca^2+^ influx^26^. Pretreatment with ILG controlled the hyperlocomotion caused by methamphetamine (METH) treatment in mice but did not cause a significant change in the monoamine levels in tissues of the cerebral cortex, striatum, nucleus acumen, thalamus, or hypothalamus^27^. METH is a psychoactive agent that increases the extracellular DA level either by suppressing DA reuptake or internalization of dopamine transporter (DAT) from the plasma membrane or stimulation of DA efflux, and results in rewarding and addictive effect^28^. Repeated administration of METH was demonstrated to affect the nigrostriatal DA pathway causing the neurodegeneration of dopaminergic terminals and subsequent reduction in tyrosine hydroxylase, DAT, and striatal DA levels, predisposing the patient to PD. Dopamine receptors (DARs) mediate various cognitive and behavioral functions associated with DA, and DA D_1_ type receptor (D_1_R and D_5_R) antagonism was found to prevent amphetamine-induced striatal neurodegeneration^29,30^. In addition, Everett et al*.* demonstrated that V_1A_R plays a substantial role to mediate the inhibitory action of oxytocin on METH-primed reinstatement and drug-seeking behaviors^31^. Thus, our present study aimed to assess the plausible role of ILG on G-protein coupled receptors (GPCRs), such as DARs (D_1_R, D_2_R, D_3_R, and D_4_R), and the vasopressin receptor (V_1A_R) via radioligand binding and functional GPCR assays.

## Results

### Inhibitory activity of ILG on recombinant human monoamine oxidases

**Figure 2:**
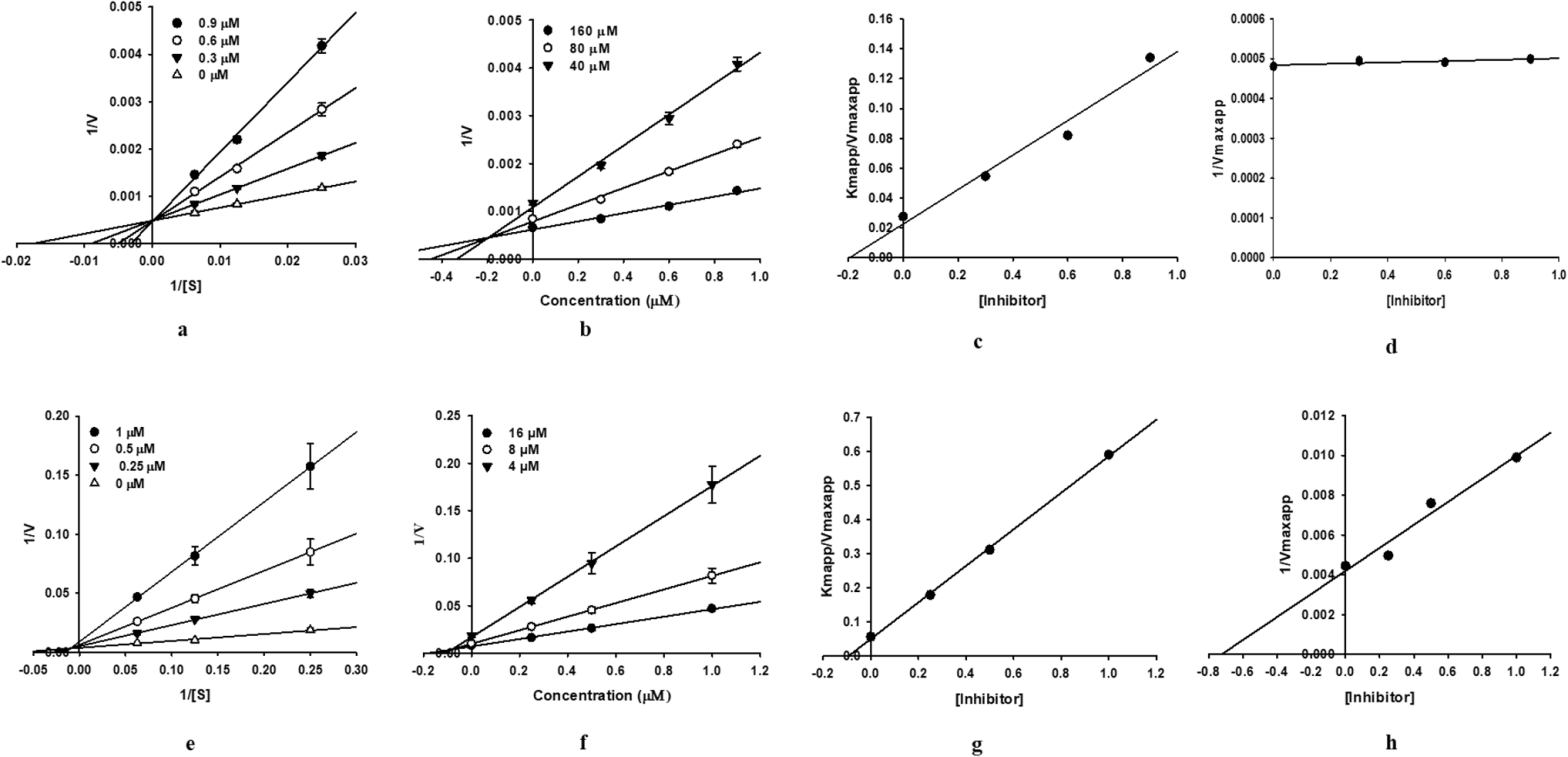
Lineweaver–Burk (**a**,**e**), Dixon (**b**,**f**), and secondary plots (**c**,**d**,**g**,**h**) of isoliquiritigenin for the inhibition of hMAO-A and hMAO-B, respectively.

ILG exhibited a strong inhibitory effect against hMAO-A and hMAO-B, as indicated by the low IC_50_ and *K*_i_ values (Table 1). ILG inhibited hMAO-A with an IC_50_ 0.68 ± 0.03 µM in a competitive manner and had a *K*_i_ value of 0.16 ± 0.00 µM. Whereas, it showed mixed inhibition of hMAO-B, with an IC_50_ 0.33 ± 0.02 µM, and *K*_ic_ and *K*_iu_ values of 0.094 ± 0.00 and 0.71 ± 0.01 µM, respectively. The modes of enzyme inhibition were determined by enzyme kinetics analyses and were represented graphically by Lineweaver–Burk, Dixon, and secondary plots (Fig. 2). The results indicated that ILG can suppress the activity of hMAO-B more efficiently than that of hMAO-A provided that a lower concentration is required to achieve the same level of activity (IC_50_ values); as *K*_ic_ < *K*_iu_, the compound has a greater affinity to inhibit unbound enzymes compared to substrate-bound enzymes.

**Table 1:** Recombinant human monoamine oxidase (hMAO) inhibitory activity of isoliquiritigenin and its enzyme kinetic parameters.

Compounds	hMAO-A			hMAO-B				SI^d^
	IC_50_^†^	*K*_i_^b^	Inhibition type^c^	IC_50_^†^	*K*_ic_^b^	*K*_iu_^b^	Inhibition type^c^	
Isoliquiritigenin	0.68 ± 0.03	0.16 ± 0.00	Competitive	0.33 ± 0.02	0.094 ± 0.00	0.71 ± 0.01	Mixed	2.06
l-Deprenyl⋅HCl^a^	13.84 ± 2.14	–	–	0.11 ± 0.002	–	–	–	125.81
Clorgyline⋅HCl^a^	0.02 ± 0.00	–	–	–	–	–	–	–

ND: not determined; (–): not tested.

^†^The IC_50_ value (μM) was calculated as mean ± standard deviation of triplicate assays.

^a^Positive control, expressed as µM.

^b^The hMAO inhibition constants (*K*_i_) were obtained from secondary plots.

^c^hMAO inhibition type was determined using Lineweaver–Burk and Dixon plots.

^d^The selectivity index (SI) was determined as the ratio of IC_50_ for hMAO-A inhibition to IC_50_ for hMAO-B inhibition.

### Computational interaction study of ILG with hMAOs

**Figure 3:**
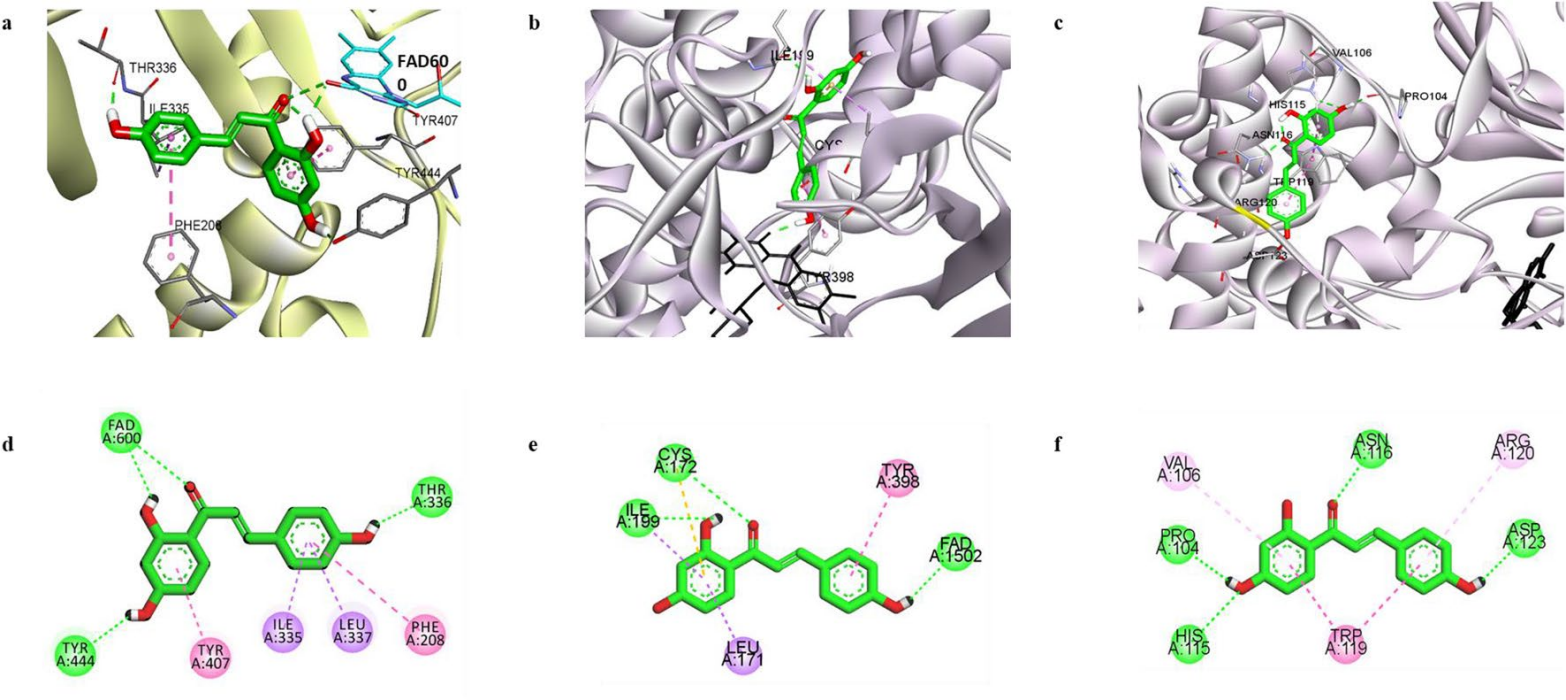
The docking pose of isoliquiritigenin (ILG) (green) at the catalytic binding site of hMAO-A (**a**). The docking poses of ILG at the catalytic (**b**) and allosteric (**c**) binding sites of hMAO-B. Key interaction molecules involved in catalytic site binding of hMAO-A (**d**) and hMAO-B (**e**) and at the allosteric site of hMAO-B (**f**) are represented by a two-dimensional binding diagram of ILG. H-bond, pi-pi-stack, pi-pi T-shaped, pi-sigma, and pi-alkyl interactions are shown with green, dark pink, violet, and light pink dashed lines, respectively.

The overall docking results including the binding energy and the interacting residues of hMAO-A and hMAO-B when ILG binds to these enzymes are shown in Table 2 and Fig. 3. To optimize and verify the docking simulation study, the reference ligand, harmine, was docked to hMAO-A (2BXR), while C17 was docked to hMAO-B (2V60). From the best pose obtained for the ligand-enzyme complex, the binding modes of ILG at active sites of the hMAO isoenzymes were visualized.

**Table 2:** Binding energy and interacting residues during the inhibition of hMAO-A and hMAO-B by isoliquiritigenin and reference ligands.

Ligands	Binding energy^a^	Interacting residues^b^	
		H-bond	Hydrophobic
**hMAO-A (2BXR)**			
Isoliquiritigenin (catalytic)	− 7.44	FAD600, Thr336, Tyr444	Ile335 (Pi-Sigma), Leu337 (Pi-Sigma), Phe208 (Pi-Pi Stacked), Tyr407 (Pi-Pi T-shaped)
HRM^c^ (Harmine)	− 6.46	FAD600	Tyr444 (Pi-Sigma), FAD600 (Pi-Sigma, Pi-Pi T-shaped, Pi-Alkyl), Tyr444 (Pi-Pi Stacked), Phe352 (Pi-Pi T-shaped), Tyr407 (Pi-Alkyl), Ile335 (Pi-Alkyl)
**hMAO-B (2V60)**			
Isoliquiritigenin (catalytic)	− 8.69	Cys172, FAD1502, Ile199, Tyr398	Ile199 (Pi-Sigma), Ile171 (Pi-Sigma), Tyr398 (Pi-Pi Stacked), Cys172 (Pi-Sulfur)
Isoloquilitigenin (allosteric)	− 8.03	Pro104, His115, Asn116, Asp123	Arg120 (Pi-Alkyl), Val106 (Pi-Alkyl), Trp119 (Pi-Pi Stacked)
Safinamide^d^	− 9.45	Ile199, Cys172, Pro102	FAD1502 (Halogen: Fluorine), Tyr398 (Pi-Pi Stacked), Tyr435 (Pi-Pi Stacked), Tyr326 (Pi-Pi T-shaped), Leu171 (Pi-Sigma), Cys172 (Pi-Sulfur)
C17^e^	− 10.60	FAD1502, Ile198	FAD1502 (Pi-Sigma), Tyr398 (Pi-Pi Stacked), Tyr435 (Pi-Pi Stacked), Tyr326 (Pi-Pi T-shaped), Leu171 (Pi-Sigma, Pi-Alkyl, Alkyl), Ile199 (Pi-Sigma, Alkyl), Leu167 (Alkyl), Phe168 (Pi-Alkyl), Cys172 (Pi-Sulfur)

^a^The estimated binding energy (kcal/mol), which signifies the binding affinity of a ligand to the active site of hMAO-A and hMAO-B.

^b^The interacting amino acid residues in the ligand-enzyme complex were determined using the AutoDock 4.2 program.

^c^Harmine, reference ligand; 7-methoxy-1-methyl-9H-pyrido[3,4-b]indole.

^d^Safinamide, reference reversible hMAO-B inhibitor; (2*S*)-2-[[4-[(3-fluorophenyl)methoxy]phenyl]methylamino]propenamide.

^e^C17, reference ligand; N7-[(3-chlorophenyl)methoxy]-2-oxochromene-4-carbaldehyde.

ILG interacted with the catalytic site residues of hMAO-A with a binding energy of − 7.44 kcal/mol, which was lower than that required for the standard ligand harmine (− 6.46 kcal/mol). The docking pose revealed that the 2′,4′-dihydroxyphenyl moiety of ILG occupied the central catalytic site of the hMAO-A and interacted with Tyr407 via a π-π T-shaped hydrophobic bond, and with Tyr444 and flavin adenine dinucleotide (FAD) via H-bonds. A carbonyl group at C1 was aligned toward FAD, which enabled its interaction with FAD through an H-bond. The aromatic ring of the 4″-hydrophenyl moiety engaged in hydrophobic interactions with active site residues Ile335, Leu337, and Phe208 through π-σ and π–π stack bonds. A H-bond between 4″-OH and Thr336 was also formed in the ILG‒hMAO-A complex.

Unlike hMAO-A, the orthosteric site structure of hMAO-B entails two cavities: a substrate cavity lined by numerous aromatic and aliphatic amino acid residues and an entrance cavity that lies adjacent to the substrate cavity. An aromatic cage formed by Tyr398 and Tyr435 along with FAD represents the recognition site for catalysis, while Tyr 326, Ile199, and Leu171 act as the key gating residues between the entrance and substrate binding cavity^23^. Molecular analysis revealed that ILG could bind with the residues of the substrate-binding cavity and the hydrophobic cavity between the substrate cavity and entrance cavity of the enzyme with a binding energy of − 8.69 kcal/mol. The 4″-hydroxyphenyl moiety of the compound interacted with Tyr398 and FAD, while another aromatic ring (2′,4′-dihydroxyphenyl moiety) was aligned to interact with gating residues Leu171 and Ile199. Pi-sulfur and H-bond interactions were formed with Cys172, a residue in the hydrophobic cavity, via 2′,4′-dihydroxyphenyl moiety and 1-CO group, respectively. In addition to catalytic interactions, ILG formed bonds with allosteric site residues of hMAO-B, with − 8.03 kcal/mol of binding energy (Fig. 3c,f). Polar H-bond associations with Pro104, His115, Asn116, and Asp123 and non-polar interactions with Val106, Trp119, and Arg120 were observed at the allosteric site.

### Receptor binding profile of ILG

The binding affinity of ILG to the human dopaminergic D_1_, D_2_, D_3_, and D_4_ receptors was measured in vitro by displacement of antagonist radioligands [^3^H]SCH23390 and [^3^H]methylspiperone from the recombinant Chinese hamster ovary (CHO)-D_1_R, human embryonic kidney (HEK)-D_2L_R, CHO-D_3_R, and CHO-D_4_R cell membranes. The V_1A_R binding property was evaluated by the agonist radioligand binding assay using [^3^H] arginine vasopressin (AVP) on the V_1A_R-transfected CHO cells and validated using AVP as a reference. Screening the binding property of ILG at 100 μM revealed significant inhibition of control-specific binding (> 50%) for D_1_R, D_3_R, and V_1A_R and moderate inhibition of D_4_R with 44.8% inhibition of control-specific binding (Table 3). High radioligand displacement was observed on the CHO-D_1_R membrane (96.0%), whereas no binding affinity was seen on the HEK-D_2L_R membrane.

**Table 3:** Human dopamine D_1_, D_2_, D_3_, and D_4_ and vasopressin V_1A_ receptor binding data of isoliquiritigenin.

Receptors	Radioligand	% Inhibition of control-specific binding^a^	Reference antagonist	Reference IC_50b_
D_1_	[^3^H]SCH23390	96.0	SCH23390	0.5
D_2L_	[^3^H]methylspiperone	21.3	(+)Butaclamol	2.6
D_3_	[^3^H]methylspiperone	52.2	(+)Butaclamol	4.1
D_4_	[^3^H]methylspiperone	44.8	Clozapine	93
V_1A_*	[^3^H]AVP	60.8	[d(CH_2_)_5_^1^,Tyr(Me)_2_]-AVP	1.4

*Agonist binding.

^a^Values are presented as the mean of the percent inhibition of control specific binding by 100 µM isoliquiritigenin performed in duplicate.

^b^The IC_50_ value of the reference antagonist (nM).

### Modulatory action of ILG on D_1_, D_3_, and V_1A_ receptors

**Figure 4:**
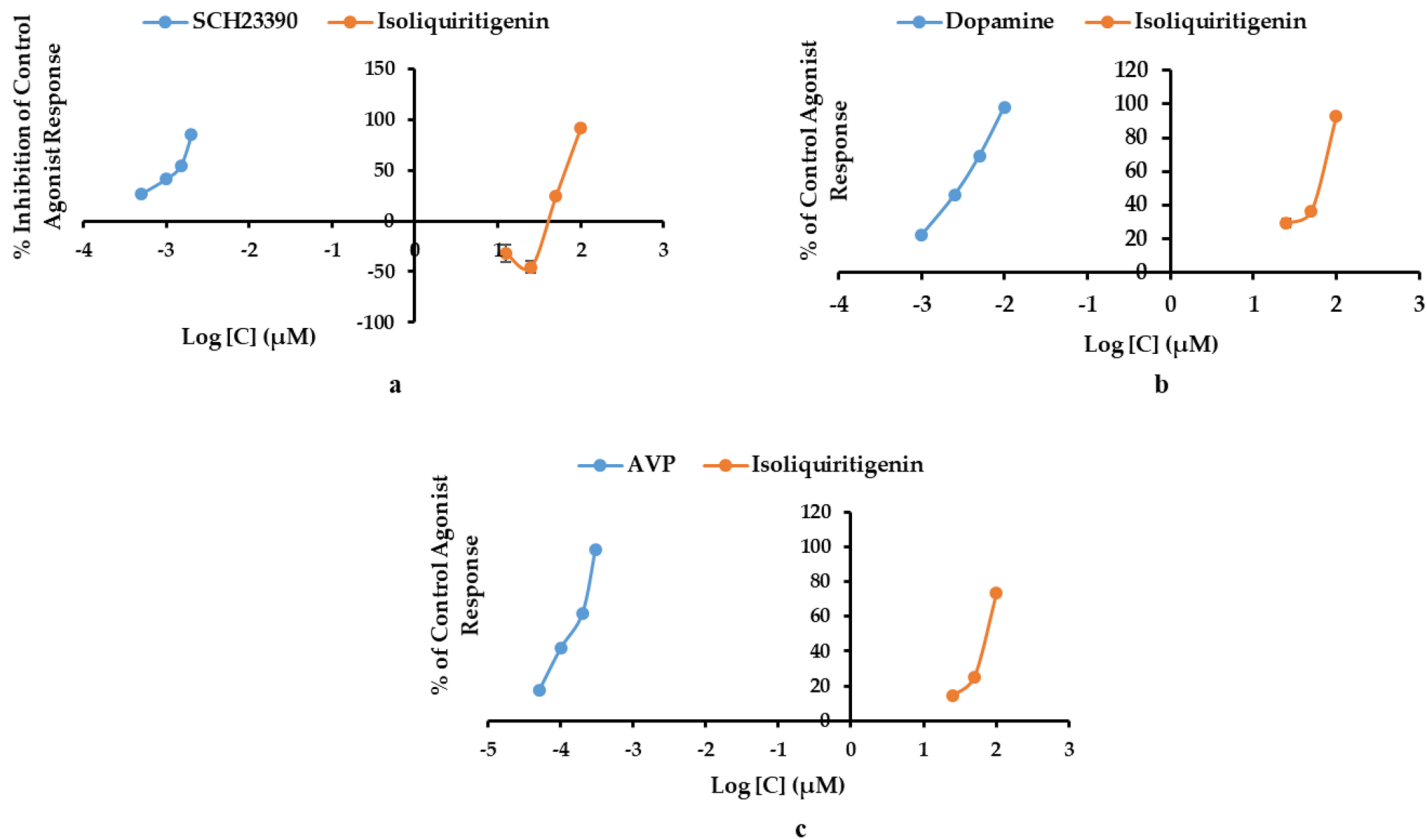
Dose–response curves of isoliquiritigenin as a dopamine D_1_R antagonist (**a**), a D_3_R agonist (**b**), and a V_1A_R agonist (**c**).

The preliminary in vitro binding screening using 100 μM ILG indicated a high binding affinity for the D_1_R, D_3_R, and V_1A_R receptors. Since binding assays are limited to determining the affinity and selectivity of ligands to receptors, we conducted cellular functional assays to reveal agonist or antagonist behavior of ILG on D_1_R, D_3_R, and V_1A_R. Functional GPCR assays showed that ILG is a D_1_R antagonist and a D_3_R and V_1A_R agonist. As shown in Table 4, 100 µM ILG inhibited the D_1_R control agonist response by 91.9 ± 3.1% and stimulated D_3_R and V_1A_R by 92.7 ± 0.5% and 73.3 ± 4.0% of the control agonist response, respectively. Figure 4 shows the dose–response curve obtained as the percent inhibition of the control agonist response on D_1_R and the percent of the control agonist response on D_3_R and V_1A_R. The concentration required to produce 50% of agonist response (EC_50_) or antagonist response (IC_50_) of ILG along with those of the reference drugs on the tested receptors are presented in Table 4. As a D_1_R antagonist, ILG had an IC_50_ value of 68.9 ± 0.3 µM, whereas, for D_3_R and V_1A_R stimulation, the EC_50_ values were 62.3 ± 0.2 and 78.6 ± 2.7 µM, respectively.

**Table 4:** Functional effect (% stimulation and % inhibition) and efficacy (EC_50_ and IC_50_) of isoliquiritigenin on human dopamine (D_1_ and D_3_) and vasopressin(V_1A_) receptors.

Receptors	% Stimulation^a^ (% Inhibition)^b^	EC_50_^c^ (IC_50_)^d^	Reference agonist^e^ (reference antagonist)^f^	Reference EC_50_^g^ (IC_50_)^h^
D_1_	− 0.5 ± 2.1 (91.9 ± 3.1)	68.9 ± 0.2	Dopamine (SCH23390)	44 (1.3)
D_3_	92.7 ± 0.5 (− 6.5 ± 5.2)	62.3 ± 0.2	Dopamine (( +)Butaclamol)	2.9 (17)
V_1a_	73.3 ± 4.0 (104.6 ± 1.8) AGO	78.6 ± 2.7	Vasopressin ([d(CH_2_)_5_^1^, Tyr(Me)_2_]-AVP)	0.46 (2.3)

^a,b^The % stimulation and the % inhibition denote the percentage of control agonist response and the percentage inhibition of the control agonist response by isoliquiritigenin at 100 µM.

^c^The concentration producing a half maximal response (µM).

^d^The concentration producing half maximal inhibition of the control agonist response (µM).

^e^^,f^Reference agonists and reference antagonists used in the assay.

^g^The EC_50_ value of the reference agonist (nM).

^h^The IC_50_ value reference antagonist (nM). AGO: The test compound induced at least 25% agonist or agonist-like effects at this concentration.

### Computational interaction study of ILG with D_1_, D_3_, and V_1A_ receptors

**Figure 5:**
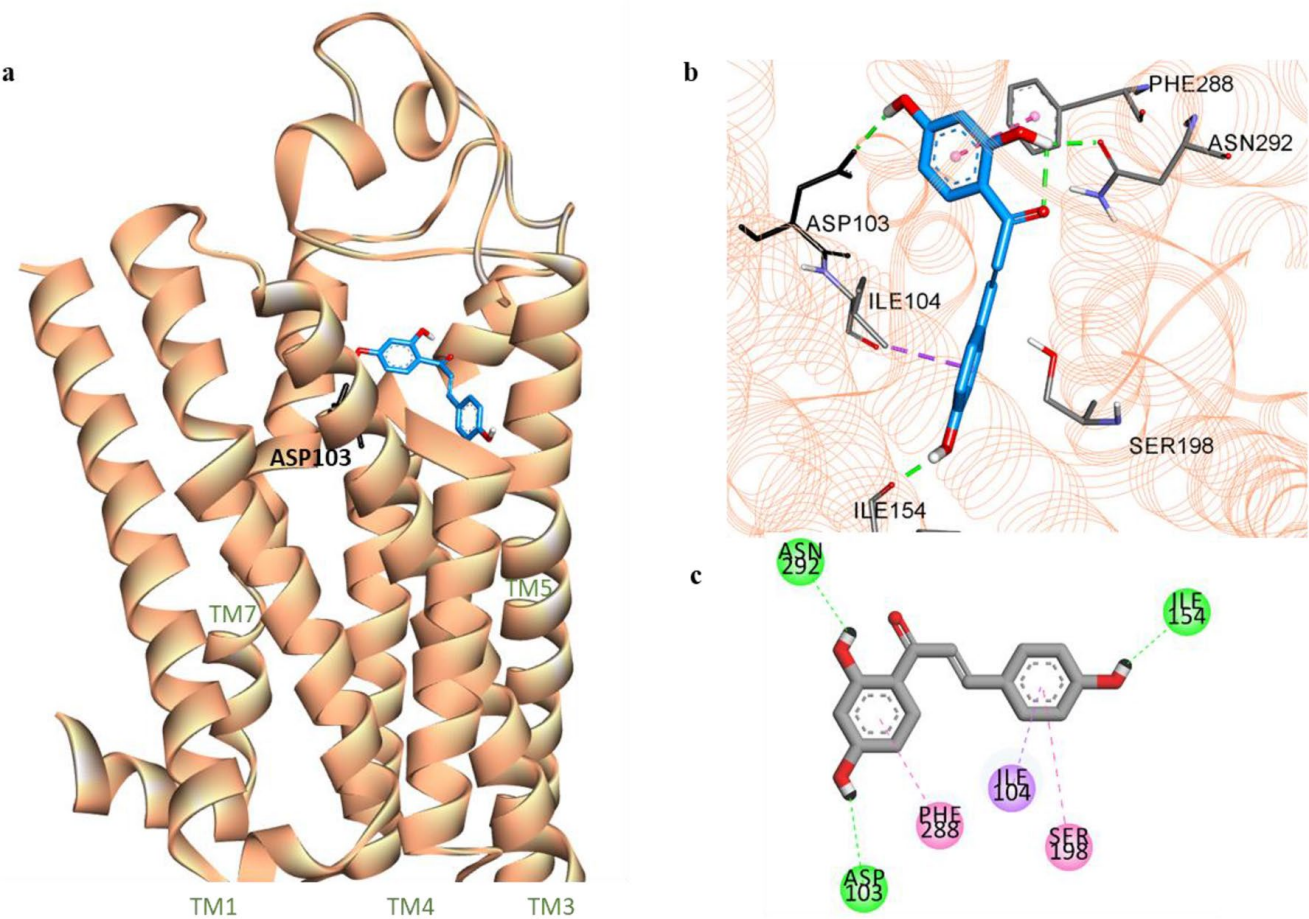
Molecular docking of isoliquiritigenin (ILG) (blue stick) to hD_1_R model (**a**). Close-up view of ligand binding pocket of hD_1_R model in complex with ILG (**b**). Two-dimensional binding diagram showing interactions of ILG-hD_1_R complex (**c**). H-bond, pi-pi-T-shaped/amide-pi stacked, and pi-sigma interactions are shown with green, dark pink, and violet dashed lines, respectively.

**Figure 6:**
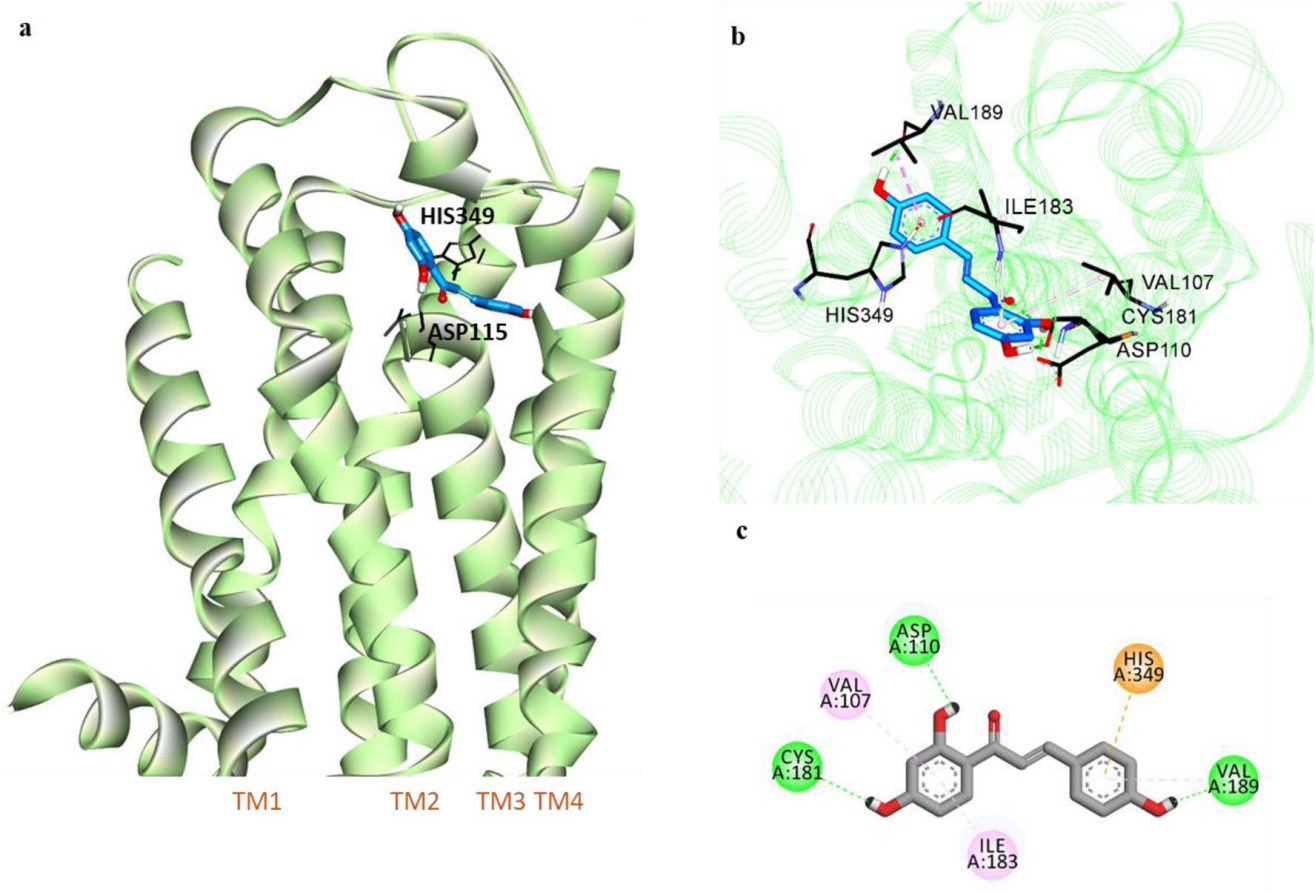
Molecular docking of isoliquiritigenin (ILG) (blue stick) to hD_3_R (**a**). Close-up view of ligand binding pocket of hD_3_R in complex with ILG (**b**). Two-dimensional binding diagram showing interactions of ILG-hD_3_R complex (**c**). H-bond, pi-alkyl, and pi-cation interactions are shown with green, light pink, and orange dashed lines, respectively.

**Figure 7:**
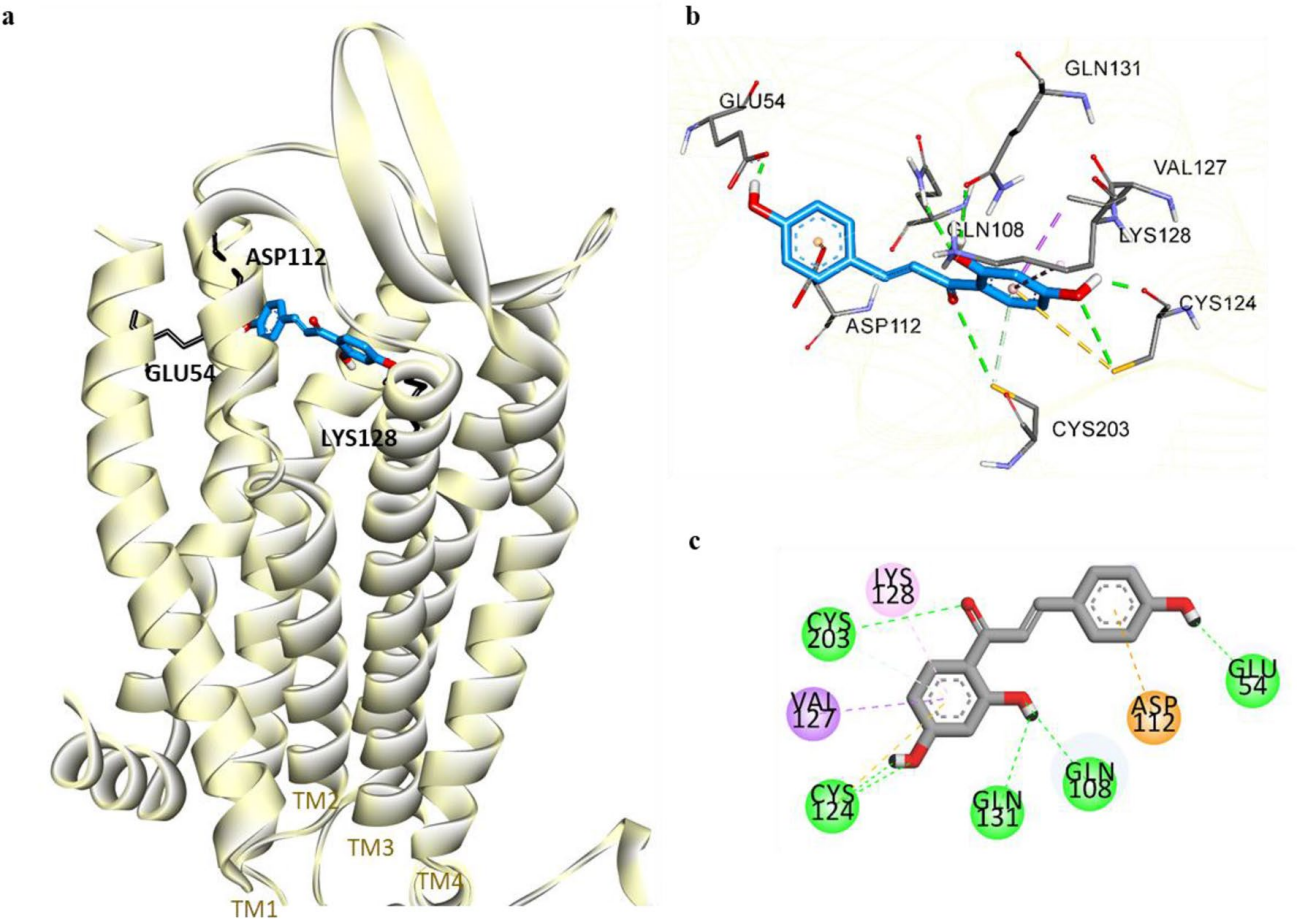
Molecular docking of isoliquiritigenin (ILG) (blue stick) to hV_1A_R (**a**). Close-up view of ligand binding pocket of hV_1A_R in complex with ILG (**b**). Two-dimensional binding diagram showing interactions of ILG-hV_1A_R complex (**c**). H-bond, pi-sigma, pi-alkyl, and pi-cation interactions are shown with green, purple, light pink, and orange dashed lines, respectively.

To examine the possible interactions of ILG at the binding sites of D_1_R, D_3_R, and V_1A_R, computational docking analyses were carried out using AutoDock 4.2. Figures 5, 6, and 7 show the docking poses of ILG within the helices of D_1_R, D_3_R, and V_1A_R, respectively. Overall ligand-receptor interactions, including H-bonding, electrostatic and hydrophobic interactions, and binding scores, obtained from the docking of the receptors with the ILG and the reference ligands are presented in Table 5. ILG was located within the ligandbinding site of a human model of D_1_R (hD_1_R) and interacted with residues Asn292, Asp103, and Ile154 through H-bonds to three hydroxyl groups at C2′, C4′, and C4″ of ILG. Two phenyl rings of the chalcone moiety anchored with conserved residues Ile104, Phe288, and Ser198 of transmembranes (TM) 3, 6, and 5, respectively, via strong hydrophobic connections.

**Table 5:** Binding energy and interacting residues of isoliquiritigenin and reference ligands at the active sites of human dopamine and vasopressin receptors (hD_1_R, hD_3_R, and hV_1A_R).

Ligand	Binding energy (kcal/mol)	Binding interacting residues		
		H-bond	Electrostatic	Hydrophobic
**hD**_**1**_**R**				
Isoliquiritigenin	− 6.12	Ile154, Asp103, Asn292	–	Ile104 (Pi-Sigma), Phe288 (Pi-Pi T-shaped), Ser198 (Amide-Pi Stacked)
Dopamine^a^	− 6.68	Asp103 (Salt bridge, OH bond), Ser202, Ser199, Ser198	Phe288 (Pi-Cation)	Phe289 (Pi-Pi T-shaped), Ile104 (Pi-Alkyl)
SCH23390^a^ (Antagonist)	− 7.16	Asp103 (Salt bridge), Ala195, Ser199	–	Le104 (Pi-sigma), Leu190 (Pi-Sigma, Alkyl), Phe288 (Pi-Pi T-shaped), Ala195 (Alkyl, Pi-Alkyl), Leu295 (Alkyl)
**hD**_**3**_**R**				
Isoliquiritigenin	− 7.24	Val189, Cys181, Asp110	His349 (Pi-Cation)	Val189 (Pi-Alkyl), Val107 (Pi-Alkyl), Ile183 (Pi-Alkyl)
Dopamine^a^	− 5.47	Asp110 (Salt bridge, O–H bond), Tyr373, Ser192	–	Val111 (Pi-Alkyl), Cys114 (Pi-Alkyl)
**hV**_**1A**_**R**				
Isoliquiritigenin	− 7.03	Gln108, Cys124, Cys203, Gln131, Glu54	Asp112	Val127 (Pi-Sigma), Lys128 (Pi-Alkyl)
AVP^a^	− 5.98	Gln311, Ser314, Asp112, Glu54	Lys128	Phe117 (Pi-Alkyl), Trp204 (Pi-Alkyl), Lys128 (Pi-Alkyl)

^a^Reference ligands for hD_1_R, hD_3_R, and hV_1A_R.

Molecular docking of ILG with D_3_R revealed that ILG can bind to the orthosteric binding site (OBS) of D_3_R with a lower binding energy (− 7.24 kcal/mol) than that required for the reference agonist DA (− 5.47 kcal/mol). Polar interaction of 4′-OH was found with Asp110 of TM3, which was similar to the case of DA but without the formation of a salt bridge. Major interactions were observed with the amino acid residues of TMs 3 and 5, such as Val107 (via π-alkyl bonding to the 2′,4′-dihydroxyphenyl ring) and Val189 (via H-bond to 4′-OH and π-alkyl bonding to the 4″-hydroxyphenyl ring). An electrostatic π-cation linkage with the key OBS residue His348 was formed with the 4″-hydroxyphenyl moiety. Moreover, ILG extends toward the second extracellular loop (ECL2) and binds to Cys181 via a H-bond and to Ile183 via a non-polar bond.

Similarly, ILG was predicted to be buried within helices 1, 2, and 3 of V_1A_R with a binding energy of − 7.03 kcal/mol, which is lower than that required for AVP binding (− 5.98 kcal/mol). In silico docking displayed five hydrophilic connections of ILG with the residues of V_1A_R that were assisted by the 2′,4′,4″-hydroxy functional groups and a carbonyl group at C1. An electrostatic interaction of the 4″-hydroxyphenyl moiety with a non-conserved hydrophilic residue (Asp112 of TM2) was observed. Hydrophobic interactions of the 2′,4′-dihydroxy phenyl ring of ILG occurred with the Val127 and Lys128 residues of TM3. Of all molecular interactions, Glu54, Asp112, and Lys128 were the common binding residues compared with the reference agonist AVP.

### Prediction of the drug-likeness and ADMET profile of ILG

Pharmacokinetic parameters and toxicity of ILG were predicted using SwissADMET and pkCSM applications. SwissADMET showed drug-likeness and lead-likeness of ILG with a lipophilicity of 2.37 (log Po/w) and a solubility of − 3.06 (soluble). A pkCSM application indicated high intestinal absorption (HIA) of ILG (> 90%) along with its probability to cross the blood brain barrier (BBB) and reach the CNS. It also predicted ILG as a safe drug-like molecule with no toxicity to hepatocytes or upon Ames testing (Table 6).

**Table 6:** Prediction of the pharmacokinetic parameters and toxicity of isoliquiritigenin.

Compound	Drug-likeness	Lead-likeness	Log Po/w^a^	Solubility^b^	HIA^c^	BBB permeability^d^	CNS permeability^e^	AMES toxicity	Hepatotoxicity
Isoliquiritigenin	Yes	Yes	2.37	− 3.06	91.096%	Yes (− 0.717)	− 2.205	No	No

^a^Octanol-water partition coefficient.

^b^LogS scale: Insoluble < − 10 < Poorly < − 6 < Moderately < − 4 < Soluble < − 2 < Very < 0 < Highly.

^c^Human intestinal absorption: If < 30%, poorly absorbed.

^d^Log BB > 0.3 considered to readily cross the blood brain barrier, while log BB < − 1 considered to be poorly distributed to the brain.

^e^Log PS values > − 2 are considered to penetrate the CNS, while log PS values < − 3 are considered unable to penetrate the CNS.

## Discussion

Of the multiple kinds of phytochemicals, flavonoids have been known to possess significant pharmacological action against different NDDs, including AD and PD^32^. ILG is a plant-derived chalcone that has shown promising effects against NDDs on multiple investigations^9^. Though earlier records of the MAO inhibition potential of ILG are available, we re-evaluated the human recombinant MAO inhibition potential of ILG obtained from *Glycyrrhizae* radix together with the investigation of enzyme kinetic parameters and the computational docking study to establish its mechanism of action.

In the in-vitro recombinant hMAO inhibition assay, ILG exhibited a remarkable inhibition of both hMAO-A and hMAO-B, with respective IC_50_ values 0.68 ± 0.03 and 0.33 ± 0.02 µM. This activity is significantly higher than the formerly reported activity by Zhou et al*.* at 61 times more potent against hMAO-A and 66 times more potent against hMAO-B. Nonetheless, in both of these studies, we found an approximately twofold higher selectivity of ILG for hMAO-B than for hMAO-A. Mohamed and colleagues had earlier investigated hMAO inhibition of ILG isolated from *Colvillea racemosa* and found selective hMAO-B inhibition with an IC_50_ value of 0.51 ± 0.01 µM, while that for hMAO-A inhibition was 22.66 ± 1.84 µM^33^. The variations in the potency of ILG for hMAO inhibition might have occurred due to different experimental conditions and procedures. To validate our results, we used the standard hMAO-A inhibitor clorgyline⋅HCl and the hMAO-B inhibitor l-deprenyl⋅HCl.

Although reports on hMAO inhibition by ILG are available, a detailed study on the mechanism of hMAO inhibition is lacking. Thus, our study incorporated an enzyme kinetics study of ILG for inhibition of hMAO isoenzymes and demonstrated concordant inhibition constant (*K*_i_) values with respect to the IC_50_ values for each enzyme (Table 1). In a previous study, ILG showed non-competitive inhibition of rMAO-A and mixed inhibition of rMAO-B^21^. The present study found competitive inhibition of hMAO-A by ILG along with a similar mode of action against hMAO-B as in rMAO-B. Though rat and human MAO-A have a 92% similar sequence identity, crystallographic analysis has revealed that a significant structural difference between hMAO-A and rMAO-A exists in the conformation of residues 210–216, which are vital for the structure of the hMAO-A active site. Unlike hMAO-A, rMAO-A is a dimer and has a smaller substrate cavity. A recent study of hMAO inhibition found liquiritigenin (LG) to have very potent action against hMAO isoenzymes compared to its activity against rMAO isoenzymes^34^. In addition, screening of coumarin and 5H-indeno[1,2-c]pyridazin-5one derivatives for inhibition of MAOs of human and rat showed greater effectiveness of compounds against hMAO-B than against rMAO-B and no correlation between human and rat pIC_50_ values^24,35^. These findings support that results drawn from experimentation in one animal model cannot consistently be extrapolated to humans.

Our docking study showed good proximity of ILG toward the isoalloxazine of the FAD cofactor in hMAO-A and hMAO-B. ILG bonded with key residues of the hMAO isoenzymes through multiple polar and non-polar interactions that resulted in strong inhibition. In hMAO-A, the 2′,4′-dihydroxyphenyl ring was engaged in interaction with the major active site residues FAD, Tyr444, and Tyr407, while in the case of hMAO-B, the 4-hydroxyphenyl moiety was responsible for forming strong interactions with FAD and a catalytic residue Tyr398. The carbonyl oxygen of ILG assisted in the formation of a stable MAO-ILG adduct through H-bonding with important active site factors (FAD in hMAO-A and Cys172 in hMAO-B). LG, a structurally interrelated flavanone form of ILG, was reported to have strong inhibition of hMAO-A and hMAO-B, with IC_50_ values of 0.27 ± 0.04 and 0.098 ± 0.00 µM, respectively. Although these IC_50_ values suggest LG to be more potent than ILG, both compounds exhibited a greater affinity for hMAO-B as the binding energy for the hMAO-B was lower than that for hMAO-A^34^. 3-Deoxysappanchalcone, a 2′-methoxy-4′,4″-dihydroxychalcone, is related structurally to ILG and demonstrates a potent hMAO inhibition potential similar to that of ILG (IC_50_ values: 10.11 and 0.68 µM for hMAO-A and hMAO-B, respectively) with a higher affinity for hMAO-B (− 8.90 kcal/mol)^36^. Other natural chalcones, such as 4-hydroxyderricin, 2,2′-dihydroxy-4′,6′-dimethoxychalcone, and broussochalcone, also have notable hMAO inhibition potential^36^.

In vitro and in vivo studies have shown that ILG can protect dopaminergic neurons from METH^37^, 6–OHDA^38^, and glutamate-induced neurotoxicity^39^ and alleviate cognitive impairment^12,14^, anxiety, and locomotor sensitization^40^. Since the modulation of DA and vasopressin receptors has been found to affect cognition, anxiety, and neuronal survival, we evaluated the binding affinity of ILG to the D_1_R, D_2_R, D_3_R, D_4_R, and V_1A_R receptors to determine whether ILG can target these receptors and identified significant binding to D_1_R, D_3_R, and V_1A_R. The functional GPCR assay showed ILG to have D_1_R antagonist and D_3_R and V_1A_R agonist properties. A ligand-D_1_R interaction study revealed that ILG can bind with the active site residues Asp103, Ile104, and Phe288 similar to the reference antagonist SCH23390. It has been reported that interactions with Asp103, Asn292, and Ser198 residues are vital for strong binding affinity of the ligand to the D_1_R^41^. The molecular docking pose of D_3_R with ILG indicates that ILG extends between helices 3, 5, and 6 to the ECL2. Some ECL2 residues (182–185) form a part of the ligandbinding pocket of D_3_R, and interactions with ECL2 residues have been acknowledged for their selectivity of ligands between D_2_ and D_3_ receptors^42^. Thus, the binding of ILG with Cys181 and Ile183 of ELC2 might be responsible for the higher affinity of ILG for D_3_R compared to D_2_R. Cotte et al*.* reported that aromatic residues in V_1A_R play an insignificant role in the agonist’s binding affinity; however, the conserved hydrophilic residues Glu108, Lys128, and Gln185 contribute significantly to the V_1A_R agonist AVP binding^43^. The H-bonding with Gln108 and non-polar linkage with Lys128, along with other known agonist binding residues (such as Glu54, Cys203, Asp112, and Val127)^44^, were detected by computational docking of ILG with a 3D model of human V_1A_R.

For treatment of PD, l-Dopa is the gold standard therapy; however, prolonged use of l-Dopa causes dyskinesia and dystonia. DAR agonists stimulate the postsynaptic DARs and enhance the function of the DA system. Thus, they are considered the first choice in *de nova* patients to delay the onset of l-Dopa therapy^45^. Another pharmacological principle used to alleviate DA deficiency in PD is the use of reversible and irreversible hMAO-B inhibitors, such as selegiline, rasagiline, and safinamide. Clinical trials have shown improvement of motor symptoms when safinamide was used as an adjunct to l-Dopa or single DA receptor agonist therapy^46^. MAO-B as well as MAO-A inhibitors have neuroprotective functions since they suppress the oxidation of monoamines and prevent the resulting generation of ROS and neurodegeneration in NDDs, such as PD and AD. MAO-A inhibitors such as iproniazid and tranylcypromine are clinically indicated for depression. Moreover, MAO-A inhibition shows potential protective roles in cancer and myocardial damage^47^.

To treat the motor and non-motor complications of PD that can arise from fluctuations in l-Dopa concentration in the plasma and the pulsatile stimulation of DARs, DAR agonists can be used as an add-on therapy to l-Dopa^48^. The D_2_/D_3_ receptor agonist pramipexole was found to improve depression in PD, anhedonia, and motor deficits^49^. D_1_R activation has shown improvement in hippocampal neurogenesis and anxiolytic and antidepressant-like effects in a rat model of PD, whereas the D_1_R antagonist SCH23390 was found to inhibit stimulant-induced DAR supersensitivity^50^. Addictive psychostimulants like cocaine, amphetamines, and METH increase D_1_R activation, leading to the amplification of c-fos and fosB expression in the striatum. These elevations in c-fos and fosB along with the METH-induced increases in c-Jun, JunB, JunD, and Fra2 expression were all inhibited by pretreatment with the D_1_R antagonist SCH23390^51^. Although the expression of D_1_Rs is reduced in PD, increased stimulation of D_1_R signaling pathways has been linked to l-Dopa-induced dyskinesia^52^. The activation of D_1_Rs by METH is known to cause neurotoxicity of nigrostriatal dopaminergic neurons, and the deletion of D_1_Rs or D_1_R antagonism considerably protected against METH-induced neurotoxic effects^53^. Thus, the neuroprotective effects of ILG, which had previously been shown to impede METH-induced neurotoxicity^37^ and hyperlocomotion without alterations in monoamine levels^27^, can be attributed to its D_1_R antagonist behavior and MAO-inhibition potential.

Former investigations in neuroscience have reported that D_3_R agonists (such as 7-OH-DPAT) can restore nigrostriatal integrity by inducing neurogenesis and therefore improve locomotor function in PD models^54^. Piribedil, another D_3_R agonist, was found to be effective in enhancing learning and memory in an animal model of cerebral ischemia-reperfusion^55^. Vasopressin V_1A_R agonist activity has been associated with cognitive function and social recognition, while V_1A_R antagonists have an anxiolytic effect. Studies conducted on V_1A_R knockout mice have identified abnormalities in social interaction and recognition on social behavior tests and reduced anxiety levels in elevated plus-maze and marble-burying behavior tests^56,57^. Therefore, the modulatory effects of ILG on V_1A_R might be related to reduced anxiety-like behavior, which was observed following ILG treatment in nicotine-withdrawn mice^40^.

The computational prediction of pharmacokinetic parameters such as drug-likeness, solubility, absorption, and CNS permeability for ILG shows favorable characteristics. In silico prediction also reveals no toxicity of ILG on AMES test and hepatotoxicity assessment. Experimentally, ILG has been found to be hepatoprotective^58^ and induces Nfr2-dependent detoxification genes^59^. Licorice is reported to be used as an adjuvant agent to enhance the efficacy of other drugs, however, herb-drug interactions have also been expected with its prolonged use. ILG, as one of the phytoconstituents, has been found to inhibit cytochrome P450 enzymes such as CYP1A2, CYP2C19, CYP2C9, and CYP3A4. These enzymes are responsible for major drug metabolism, and inactivation of these enzymes can reduce drug metabolism, thus, leading to increased plasma concentration and the risks of side effects^60^. Treatment with higher doses (36 and 100 µM) of ILG inhibited follicular growth and steroidogenesis owing to the perturbation in the expression of key steroidogenesis regulators such as CYP17A1, CYP19A1, and HSD17B1^61^.

Overall, this study reinvestigated the hMAO-A and hMAO-B inhibitory activity of ILG and established the enzyme inhibition mode through kinetic assays and computational docking. Our study recognized that ILG is a potent hMAO-inhibitor showing competitive inhibition against hMAO-A and a mixed mode of inhibition against hMAO-B. ILG was observed to have a significant binding affinity for the D_1_, D_3_, and V_1A_ receptors. Probing the modulatory activity of ILG on DA (D_1_ and D_3_) and vasopressin (V_1A_) receptors revealed that ILG has an antagonist effect on the D_1_ receptor and agonist effects on the D_3_ and V_1A_ receptors. The multi-target nature of ILG together with the prediction of suitable pharmacokinetics and the toxicity profile renders this compound as a potential flavonoid for the management of PD and its related neurological symptoms. Future experimental studies on ILG’s pharmacological and toxicological properties and adverse effects will be important to confirm its therapeutic efficacy and benefits.

## Materials and methods

### Chemicals and reagents

For the recombinant hMAO inhibition assay, a MAO-A/B assay kit (Promega Cooperation, Madison, WI, USA), recombinant hMAO isoenzymes, and the standards l-deprenyl⋅HCl (Sigma Aldrich, St. Louis, MO, USA) were used. For the GPCR functional assay, reference drugs of DA, serotonin, AVP, clozapine, butaclamol, SCH23390, (S)-WAY-100635 and [d(CH_2_)_5_^1^, Tyr(Me)_2_]-AVP as well as the test compound, ILQ, were purchased from Sigma-Aldrich (St. Louis, MO, USA). Transfected CHO and human embryonic kidney (HEK-293) cell lines were generated by Eurofins Discovery (Le Bois I’Eveque, France). The cell culture media of Roswell Park Memorial Institute (RPMI-1641), Dulbecco's modified Eagle medium (DMEM) buffer, Hank’s balanced salt solution (HBSS) buffer, and 4-(2-hydroxyethyl)-1-piperazineethanesulfonic acid (HEPES) buffer were procured from ThermoFisher Scientific (USA).

### In vitro hMAO-A and -B inhibitory assay and enzyme kinetics

Experimental conditions and procedures for this experiment were as described^62^. Briefly, we added 12.5 µl of the test compound or l-deprenyl to an aliquot of 12.5 µl of beetle luciferin derivative substrate (initial concentrations of 160 µM and 16 µM for hMAO-A and hMAO-B, respectively) in each well of the plate. A 25-µl enzyme solution was added to the test samples to initiate the reaction. After an hour of incubation of the mixture at 25 °C, 50 µl reconstituted luciferin detection reagent was added to every well to stop the hMAO reaction, and an additional 20 min of incubation at 25 °C was performed. Then, the luminescence reading was taken on a FilterMax F5 Multi-Mode microplate reader (Molecular Devices, LLC., CA, USA).

The enzyme inhibition kinetics were analyzed using varying concentrations of hMAO substrate (40–160 μM for hMAO-A and 4–16 μM for hMAO-B) and ILG (0–1 μM), as shown in Fig. 2. Inhibition constants (*K*_i_) for each enzyme inhibition were obtained from secondary plots analyzed using SigmaPlot 12.0 TM software (SPCC, Inc., Chicago IL, USA).

### Radioligand binding assays

The binding assays were carried out using validated methods and standard operating procedures used by Eurofins Cerep (catalog items 0044, 1405, 0048, 0049, and 0159). For the human D_1_R, D_3_R, and D_4_R binding assays, membrane homogenates (8–80 µg) of the respective receptor-expressed CHO cells were suspended in a buffer containing 50 mM Tris–HCl, 5 mM KCl, 5 mM MgCl_2_, 1.5 mM CaCl_2_/120 mM NaCl, and 5 mM EDTA and incubated for 60 min at 22 °C with appropriate radioligand [^3^H]SCH23390 or [^3^H]methylspiperone either in the presence or absence of the test compound. The human D_2L_R binding experiments were run on membrane preparations prepared from HEK cells. The plasma membrane homogenates (40 µg) of D_2L_R-expressing HEK cells were incubated with 0.3 nM [^3^H] methylspiperone for 60 min at 22 °C after being suspended in a binding buffer containing 50 mM Tris–HCl, 5 mM MgCl_2_, 1 mM EDTA, 1 UI/mL ADA, 1 µg/ml leupeptin, 1 µM pepstatin, and 10 µg/mL trypsin inhibitor. Human V_1A_R binding was assessed using membrane homogenates of transfected CHO cells suspended in a buffer containing 5 mM Tris–HCl, 5 mM MgCl2, and 0.1% BSA and incubated with [^3^H]AVP at 22 °C for 60 min. 1 µM SCH23390 (for D_1_R), 10 µM (+)-butaclamol (for D_2L_R, D_3_R and D_4_R), and 1 µM AVP (for V_1A_R) were used to define non-specific binding.

Following incubation, the binding reaction within the samples was terminated by vacuum filtration through 0.3% polyethyleneimine-treated glass fiber filters (GF/B, Packard). The filters were washed several times with an ice-cold wash buffer (50 mM Tris–HCl) using a 96-sample cell harvester (Unifilter, Packard) and dried. Scintillation cocktail (Microscint O, Packard) was added to the dried filters, and their radioactivity was determined using a scintillation counter (Topcount, Packard). The radioligand binding results were expressed as the percent inhibition of control specific binding given by the following equation: 100 – [(measured specific binding/control specific binding) × 100].

To verify the results, the standard compounds were tested at different concentrations in each of the binding assays to obtain competition curves from which their IC_50_ values were determined. The binding affinity of ILG to the receptors was screened at 100 μM.

### In vitro functional GPCR assay

Cellular and nuclear receptor functional assays were performed at Eurofins Cerep using human recombinant CHO cells transfected with the GPCR genes of interest (D_1_R, D_3_R, and V_1A_R). The in-house assay protocol for the assay was as described in our earlier report^63^. The results of the functional assay were based on the measurement of effects on the cAMP level and calcium ion mobilization.

### Measurement of cAMP level

Stably transfected CHO cells containing the cDNA of human D_1_ and D_3_ receptors were suspended in a medium containing an HBSS buffer that had been supplemented with 20 mM HEPES buffer and 500 µM IBMX. This cell suspension was distributed into assay plates at a density of 5 × 10^3^ cells/well and incubated at 25 °C (for the D_1_R transfected cell suspension) or 37 °C (for the D_3_R transfected suspension) for 10–30 min both with and without ILG or standard. Then, the D_2_-labeled cAMP conjugate was dispensed into the cell plate followed by the addition of europium cryptate-labeled anti-cAMP antibody. A lysis buffer was added to each well, and the resulting mixture was incubated for 1 h at 25 °C. The homogeneous time-resolved fluorescence (HTRF) reading was recorded using a PerkinElmer Envision microplate reader (Waltham, MA, USA) at an excitation intensity of 337 nm and emission intensities of 620 and 665 nm. The cAMP level was calculated as the ratio of the signal measured at 665 nm to that measured at 620 nm. The final results were illustrated as the percentage of the control agonist response and as the percentage inhibition of the control agonist response.

### Measurement of intracellular calcium levels

Calcium ion influx was determined fluorimetrically to establish the functional effect of ILG on V_1A_R. Transfected CHO cells were suspended in an HBSS/20-mM HEPES buffer and distributed into microplate wells at a density of 1 × 10^5^ cells/well. A fluorescent probe (Fluo8 Direct, Invitrogen, Carlsbad, CA, USA) was mixed with probenecid in HBSS/20 M HEPES (pH: 7.4) discretely and applied to each well, and left to equilibrate with the cells at 37 °C for 60 min. Thereafter, the assay plate was kept in a microplate reader (CellLux, PerkinElmer, Waltham, MA, USA), and ILG, the reference agonist, or HBSS buffer (control) was added to the plate; finally, the fluorescence was measured. The agonist effect was determined as the percentage of the control response to 1 µM AVP, while the antagonist behavior was calculated as the percentage inhibition of the control response to 10 nm AVP.

### Homology modeling

The sequences for hD_1_R and hV_1A_R were retrieved from the UniProt database with respective IDs of P21728 (DRD1_HUMAN) and P37288 (V1AR_HUMAN). Due to the high sequence similarity in the binding site and the overall structure between DA D_1_R and β_2_ adrenergic receptor (β_2_R), the model for this experiment was derived from the template of the β_2_R crystal structure obtained from Protein Data Bank (PDB) ID 2RH1 using the Swiss-Model server^64^. In the case of hV_1A_R, a μ-opioid receptor (PDB: 4DKL) was selected for model building^65,66^. The model was constructed based on the target (hV_1a_R)-template (4DKL) alignment using the Swiss-Model server^67^. The ModRefiner server was used to refine the model^68^.

### Computational analyses

The molecular docking study was carried out using AutoDock 4.2^69^. X-ray crystallographic structures of hMAO-A, hMAO-B, and hD_3_R were attained from the RCSB PDB with IDs 2BXR, 2V60, and 3PBL, respectively. The three-dimensional chemical structure of ILG and the reference compounds were derived from the PubChem Compound database (NCBI). Discovery Studio (v17.2, Accelrys, San Diego, CA, USA) was used for protein preparation. The AutoDockTool was utilized for adding necessary parameters like Gasteiger charges and rotatable bonds to perform docking simulations. Grid maps were generated by the AutoGrid program. The protocols for both rigid and flexible ligand docking consisted of 10 independent generic algorithms. The docking pose with the lowest binding score was chosen, and the results were visualized using Discovery Studio. Internal motion of the receptor during docking performance was not considered.

### Drug likeliness and ADMET prediction of ILG

HIA, BBB, and CNS permeability, and toxicity profile were predicted using a web-based pkCSM application (http://biosig.unimelb.edu.au/pkcsm/prediction)^70^, whereas the lipophilicity, solubility drug-likeliness, and lead-likeliness of ILG were predicted by SwissADME (http://www.swissadme.ch)^71^.

### Ethics declaration

No animal model was used for the study. The study was based on in vitro enzyme and cellular experiments, and in silico study.

## Data Availability

The data relevant to this study are provided in the manuscript and additional information can be obtained from the corresponding authors upon reasonable request.
